# Integrated Strategies for *Aedes aegypti* Control Applied to Individual Houses: An Approach to Mitigate Vectorial Arbovirus Transmission

**DOI:** 10.3390/tropicalmed9030053

**Published:** 2024-02-24

**Authors:** Danielle Cristina Tenório Varjal de Melo, Eloína Maria de Mendonça Santos, Morgana Nascimento Xavier, Josimara do Nascimento, Victor Araújo Barbosa, André Luiz de Sá Oliveira, Marcos Vinícius Meiado, Maria Alice Varjal de Melo-Santos, Marcelo Henrique Santos Paiva, Gabriel da Luz Wallau, Cláudia Maria Fontes de Oliveira

**Affiliations:** 1 Department of Entomology, Fiocruz/PE, Aggeu Magalhães Institute, Recife 50740-465, PE, Brazilmaria.varjal@fiocruzl.br (M.A.V.d.M.-S.);; 2 Secretraria de Saúde Municipal de Jaboatão dos Guararapes, Nucleus of Biology, Academic Center of Vitória, Federal University of Pernambuco, Recife 50670-901, PE, Brazil; 3 Center for Statistics and Geoprocessing, Fiocruz/PE, Aggeu Magalhães Institute, Recife 50740-465, PE, Brazil; 4 Department of Biosciences, Federal University of Sergipe, Itabaiana 49100-000, SE, Brazil; 5 Life Science Center, Academic Center of Agreste, Federal University of Pernambuco, Caruaru 50670-901, PE, Brazil

**Keywords:** surveillance program, viral surveillance, integrated vector management, one health, mosquito burden mitigation

## Abstract

*Aedes aegypti* and *Culex quinquefasciatus* mosquitoes are vectors of different arboviruses that cause a large burden of disease in humans worldwide. A key step towards reducing the impact of arboviruses on humans can be achieved through integrated mosquito surveillance and control approaches. We carried out an integrated approach of mosquito surveillance and control actions to reduce populations of these insects along with a viral surveillance in a neighborhood of Recife (Northeastern Brazil) with high mosquito densities and arbovirus transmission. The actions were carried out in 40 different houses in the Nova Descoberta neighborhood. The area was divided into two groups, the control group using tools to monitor the mosquito density (1 OVT; 1 Double BR-ovt; monthly capture of alates) and the experimental group with control actions using surveillance tools in an intensified way (2 OVTs; 2 Double BR-ovts; fortnightly capture of alates; toxic baits). We evaluated the study’s impact on the mosquito density via the Egg Density (ED) and Adult Density (AD) over a period of 12 cycles of 28 days each. The collected adult mosquitoes were processed via RT-qPCR for DENV, CHIKV and ZIKV and, subsequently, the Minimum Infection Rate (MIR) was calculated. After 12 cycles, we observed a 91% and 99% reduction in *Aedes* ED and AD in the monitored properties, as well as a 76% reduction in the AD of *Cx. quinquefasciatus* in the same properties. Moreover, we detected circulating arboviruses (DENV and ZIKV) in 19.52% of captured adult mosquitoes. We show that enhancing entomological surveillance tools can aid in the early detection of possible risk areas based on vector mosquito population numbers. Additionally, the detection of important arboviruses such as ZIKV and DENV raises awareness and allows for a better selection of risk areas and silent virus spread. It offers supplementary information for guiding emergency mosquito control measures in the target area. The goal is to minimize human–vector interactions and, subsequently, to lower the risk of transmitting circulating arboviruses.

## 1. Introduction

Brazil is a tropical country with environmental characteristics that favor the abundance of mosquitoes and, consequently, the co-circulation and dissemination of multiple arboviruses [1]. Annually, 50 to 100 million people are infected worldwide, with more than 400 million people living in areas at risk of infection [2]. Inappropriate environmental conditions are factors that contribute to the high virus load in the human population, in addition to the lack of mass immunization and specific treatments for the majority of circulating arboviruses, and even their co-circulation [3,4]. The socioeconomic difficulties of the population, poor sanitary conditions and the accumulation of non-recyclable waste also contribute to the proliferation and abundance of mosquitoes that transmit these pathogens [5,6].

Between 2015 and 2016, Brazil was the epicenter of the Zika virus epidemics, raising worldwide concern due to the alarming and increasing infection cases associated with several newborn malformations (known as Congenital Zika Virus Syndrome) and neurological sequelae in adults (Guillain–Barré syndrome) [7,8]. In the following two years, there was a consistent decrease in the number of Zika and dengue notifications, which some authors linked to immunity acquired after an outbreak and a high rate of DENV infection over several years [9], and to a possible cross-immunity between these arboviruses [10,11]. However, in 2019, the human cases increased again, suggesting that cross-immunity declined through time [9]. In the first quarter of 2020, there was an upward trend in the number of dengue cases, concomitantly with the increase in the number of cases of severe acute respiratory syndrome (SARS-CoV-2) in the country, which caused the collapse of the health system. The mobilization of epidemiological surveillance teams to deal with the coronavirus pandemic (COVID-19) has resulted in a delay in registration and even underreporting of arbovirus cases [12]. Despite this scenario, nearly a million dengue cases were reported in the country over a one-year timespan (November 2019 to December 2020) [13].

Mosquitoes of the Culicidae family can transmit several arboviruses that cause debilitating and, eventually, deathly diseases in humans. Among urban mosquito vectors, two are widely distributed in Brazil: *Aedes aegypti* (Linnaeus, 1762) and *Culex quinquefasciatus* (Say, 1823) [14,15]. *Ae. aegypti* is considered one of the main global arbovirus vectors [16], transmitting a wide diversity of viruses such as DENV, ZIKV and CHIKV. This species invaded the American continent by means of merchant ships and slavers from the 16th century. In 1955, it was eliminated from Brazil, but in the mid-1980s, it was reintroduced to the country and has rapidly spread all over the country [16,17]. *Cx. quinquefasciatus* is also known to transmit arboviruses, such as West Nile Virus (WNV) and ZIKV [18] and is the only known vector of filarial worms responsible for lymphatic filariasis in the country [19]. This species is found in high densities, especially in human settlements with lower incomes. In these clusters, disordered urban growth has placed the human population in close and frequent contact with several vectors and pathogens, increasing the risk of disease outbreaks. Therefore, to reduce these risks, it is necessary to implement the continuous control and surveillance of vector-transmitted pathogens. Entomological surveillance provides the monitoring of mosquito species that occur in the region and detects the circulation of pathogens through the determination of vector infection status [20]. The early detection of arboviruses in circulating mosquitoes is essential information for making rapid decisions and deploying intensive control actions, reducing the risk of human outbreaks [21]. It is through biological transmission that arboviruses are maintained in nature, where viruses, mosquitoes and vertebrates are the essential components [22]. Thus, xenomonitoring surveillance is extremely relevant for public health, as it detects the circulation of arboviruses in a given area before possible outbreaks occur.

In Recife, the population control strategy for *Aedes aegypti* and *Culex quinquefasciatus* is mainly based on the search, elimination or treatment of breeding sites using chemical and biological larvicides [23]. This action is recommended by the National Program for the Control of Dengue and other arboviruses (PNCD) [24] and by the National Program for the Elimination of Lymphatic Filariasis (PNEFL) [25]. The early detection of arboviruses in circulating mosquitoes is essential information for making rapid decisions and deploying intensive control measures, thereby reducing the risk of human outbreaks [19,26,27]. Some tools used for mosquito surveillance and control are often combined in the field. To control the egg density, the OVTs installed by the Recife Health Department in the study area were used. Oviposition traps, such as Ovitrap [28] and Double BR-ovt [29] as examples, when associated with larvicides such as those based on *Bacillus thuringiensis* var *israelensis* (Bti), can be used as a continuous tool to interrupt the culicid life cycle and, at the same time, stimulate oviposition in these traps [30,31]. Such integrated strategies can remove hundreds of thousands of eggs from the environment, or even trapping adult mosquitoes by some glue dispositive [29]. An entomological aspirator is another tool used in surveillance that can be applied as a complementary mechanical control of adult mosquitoes, when used with high frequency, by removing them from the environment. [32].

The combination of efficient vector surveillance, control tools and resources to manage vector-borne diseases is known as Integrated Vector Management (IVM) [33]. In Pernambuco, Brazil, Regis and collaborators (2013) successfully reduced the mosquito density using IVM [26]. Other studies have also demonstrated the effectiveness of the integrated use of mosquito control methods [34,35,36]. In this study, we evaluated the impact of alternative integrated control actions on the mosquito density to verify whether there was a reduction in continuous mosquito–human contact, in individual residences, in an area with high levels of *Ae. aegypti* and *Cx. quinquefasciatus*.

## 2. Materials and Methods

*Study Area*—the city of Recife-PE, located in the Northeast region of Brazil (8°04′03″ S 34°55′00″ W), has an annual average temperature that varies between 23 °C and 31 °C, relative humidity between 70% and 90% and average annual precipitation of 1551 mm. The study monitored one area in the neighborhood of Nova Descoberta that presents a very high risk of dengue transmission, according to the *Aedes aegypti* Rapid Index Survey (LIRAa ≥ 4), conducted in 2018 and remains with this index to the present day (LIRAa 2022). The neighborhood of Nova Descoberta has an area of 180 hectares (~34,000 inhabitants). According to IBGE (2017), only 54.4% of the neighborhood has adequate sanitation. It is a neighborhood with a high density of buildings and little vegetation cover. It has an annual average temperature that fluctuates between 24 °C and 27 °C and a rainfall regime present throughout the year. In Nova Descoberta, mosquito control actions preconceived by the PNCD [24] and PNEFL [25] programs have been carried out since 2000 and 2002, respectively, with the main action being the treatment of breeding sites using Bti larvicide (VectoBac^®^ WDG) and Vectolex^®^ (ValentBioSciences). However, the entomological surveillance is related only to *Ae. aegypti* by LIRAa.

### 2.1. Tool for Monitoring Mosquitoes

*Adults*—mosquitoes were captured indoors and outdoors, using entomological aspirators, in resting places, under furniture, behind curtains, on ceilings and walls of homes [37]. The entomological aspirator is manufactured in Brazil by the company Horst Armadilhas, São Paulo, Brazil, consisting of a PVC tube, a 12-volt battery and an inverted fan to suck up mosquitoes to a collection bag (fabric). Collections were carried out in the morning from 9:00 a.m. to 12:00 p.m., lasting approximately 15 min of aspiration effort/property, starting from the right side and ending on the left side in an indoor area, covering different places, such as the living room, bedrooms, bathrooms, kitchen, service area. In the IAM-Fiocruz Laboratory, the collected mosquitoes were anesthetized via freezing, identified via species using dichotomous keys [14,38]; they were counted and sexed, and the females were stored at −80 °C for further molecular analysis to detect arboviruses.

*Eggs*—we used secondary data, provided by the Health Department of Recife, referring to the monthly entomological surveillance of *Ae. aegypti* carried out by 14 sentinel-ovitraps (S-OVTs), installed in the same area. These data served to monitor the population fluctuation of the species by estimating the density of eggs of *Aedes* spp.

### 2.2. Tools for the Mosquitoes’ Control in Different Phases

**Figure 1:**
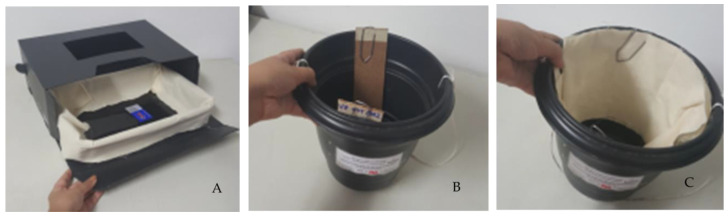
Double BR-ovt oviposition trap (**A**); Sentinel Ovitrap-OVT-S (**B**); and control Ovitrap-OVT-C (**C**). Source: Melo, 2020 [39].

*Eggs elimination*—the trap used for the massive removal of *Aedes* spp. eggs from the environment was adapted to the model of Regis et al. [32]. The first ovitrap model (OVT) consists of a black plastic container with a capacity of 2.5 L of tap water and two different supports, one made of wood (5 cm × 15 cm) 5 cm long by 15 cm wide or a piece of cotton fabric measuring (20 cm × 60 cm) 20 cm long by 60 cm wide, fixed inside the pot with clips, like two different substrates for oviposition. The OVTs were installed outdoors, at least 2 or 3 m apart, in the peridomestic area (Figure 1) [29]. And for *Culex* spp., the trap used for the massive removal of eggs from the environment was adapted to the model used by Xavier et al. (2020) [29]. The BR-ovt Duo [29] was composed of a black polyethylene box measuring (24 cm × 35 cm × 13 cm) 24 cm long by 35 cm wide and 13 cm high, which has a central opening measuring 16 cm long by 9 cm wide at the top. In this trap, a black plastic container (4 L) was placed inside the box, with its inner wall covered by a strip of cotton fabric measuring (20 cm × 110 cm) 20 cm long and 110 cm wide, also serving as a substrate for laying Aedes spp. eggs. For operational reasons, the adhesive edge was not used to collect adults from the Double BR-ovt trap. 1 g of VectoMax^®^ biological larvicide was added to all traps (OVT and Double BR-ovt) to prevent the traps from becoming breeding sites. Trap maintenance was carried out every 28-day cycle, when the operator collected the oviposition substrates and replaced them with new ones, water and larvicide. At each maintenance, these substrates were placed in duly identified plastic bags and taken to the laboratory. Before counting eggs, the substrates were kept at room temperature under a screen to dry completely. All *Aedes* eggs present in the substrates were counted under a stereoscopic microscope with a 10-fold increase in size.

The eggs were not identified according to the species level for operational reasons, but in the study area, through historical data and the capture of adults, we only detected the presence of *Ae. aegypti*.

Treatment of breeding sites for larval elimination—a real or potential breeding site found in each property was treated, every 28 days (cycle), with the biological larvicide VectoMax^®^ (ValentBioSciences, 2.7% Lsp and plus 4.5% Bti), at 5.5 g/m^2^, according to the manufacturer’s instructions. At the same time, the containers with potable water were treated with Bti, VectoBac^®^ WG (ValentBioSciences, 37.4%), at 0.2 g/100 L, such as preconized by the PNCD.

*Adult mosquito elimination*—two different strategies were employed for the mosquitoes’ elimination: the first one was the use of aspiration to remove mosquitoes from the environment mechanically, using the methodology described for monitoring action, in biweekly or monthly intervals for 12 cycles, according to the intervention plan. Another strategy used was the use of toxic sugar baits (TSBs) [38], based on the attract to kill technique; they were used in some residences for 7 consecutive cycles. The baits were prepared with cotton wool soaked in a sucrose solution (10%), treated with Ivermectin (0.05%), stored in acrylic containers with a capacity of 25 mL [39]. The TSBs were attached to the ovitraps for potentializing the contact with the females and the chances to compete with other natural sources of carbohydrate in the environment. The replacement of TSBs was carried out at each cycle.

**Figure 2:**
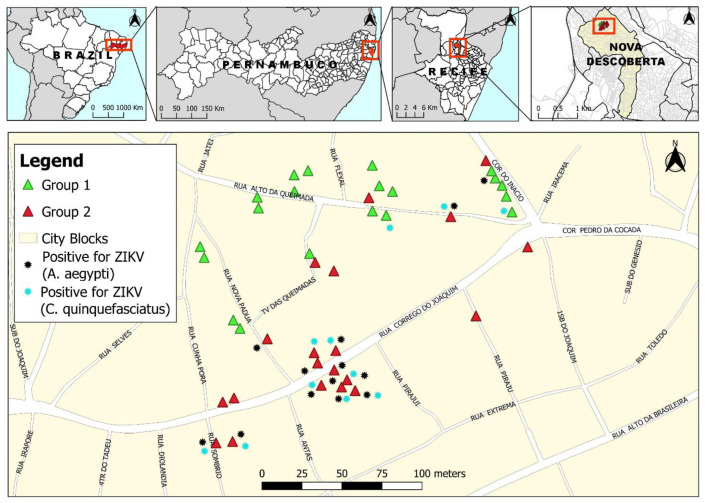
Area map with experimental design carried out in the study. Group 1—simple actions with the installation of two oviposition traps (one OVT and one Double BR-ovt) and capture by aspiration of adult mosquitoes monthly. Group 2—double actions with the installation of four oviposition traps (two OVTs and two Double BR-ovts), capture of adult mosquitoes by aspiration fortnightly and use of toxic sugar baits (TSBs) attached to oviposition traps. Black stars correspond to properties with *Ae. aegypti* mosquitoes positive for ZIKV, and blue stars *Cx. quinquefasciatus* mosquitoes also positive for this virus. In the study area (Nova Descoberta), *Aedes aegypti* was also monitored by sentinel-ovitraps (S-OVTs) as part of entomological surveillance from the Health Department of Recife. Source: the authors, from QGIS software version 3.16.

*Intervention Plans in the Study Area*—in the area, forty properties were selected (Figure 2) and two groups were established, with the same number of properties (20), with different plans of actions to control mosquitoes, implemented from January to December 2018. In the properties of group 1, corresponding to a simple action, were placed 1 outdoor OVT, 1 indoor double BR-ovt and a monthly aspiration of adult mosquitoes inside and outside the residences. In group 2, corresponding to double actions, were placed 2 outdoor OVTs, 2 double internal BR-ovts, biweekly mosquitoes’ aspiration and furthermore, from July to December 2018, toxic sugar baits (TSBs) attached with oviposition traps. The mosquito densities were monitored for both groups, for 12 cycles.

*Virus Surveillance*—collected mosquitoes were separated into pools of up to 10 females/property and submitted to molecular detection experiments for DENV, CHIKV and ZIKV viruses. **Preparation of sample homogenate and RNA extraction:** Mosquitoes were organized in pools and placed in 1.5 mL microtubes. A total of 300 µL of ultra-pure water (Invitrogen^®^) was added to each pool, and, with the aid of autoclaved pistils, mosquitoes were macerated. After sample homogenization, the macerate was centrifuged at 4000 rpm for 4 min. Aliquots of 100 µL of the supernatant were separated for each sample for RNA extraction. RNA was extracted with TRIzol^®^ (Invitrogen^®^), produced by Thermo Fisher Scientific, Waltham, Massachusetts, USA following the manufacturer’s protocol. **RT-qPCR reaction:** RNAs from *Ae. aegypti* and *Cx. quinquefasciatus* pools were submitted to a singleplex RT-qPCR (Table S1) to detect ZIKV, using primers described elsewhere [40]. *Aedes aegypti* samples were also analyzed using an RT-qPCR duplex (Table S2) to detect DENV and CHIKV [41,42]. Samples (up to 20 pools/cycle of *Ae. aegypti* and *Cx. quinquefasciatus*) were analyzed using specific probes for each researched target. Primers and probes are described in Table S3. In RT-qPCR, the QuantiNovaProbe RT-PCR kit (QIAGEN^®^) was used, and the reactions followed these conditions: 45 °C for 15 min, 95 °C for 5 min, followed by 45 cycles of 95 °C for 5 s and 60 °C for 45 s on QuantStudio^®^5 equipment (AppliedBiosystems/Thermo Fisher Scientific, Waltham, MA, USA). All samples were tested in duplicates, with negative controls (only the mix of RT-qPCR, no RNA sample) and positive controls for all viruses (viral library of the entomology department of IAM—PE). A description of reagents used for *Aedes aegypti* and *Culex quinquefasciatus* samples in RT-qPCR reactions for the detection of ZIKV, DENV and CHIKV is available in Supplementary Materials Tables S1–S3.

*Statistical Analysis—Minimum Infection Rate (MIR) Calculation:* to calculate the viral infection rates in the collected samples, the MIR—Minimum Infection Rate—was used. This index is calculated by dividing the number of infected pools by the total number of mosquitoes tested, multiplied by 1000 [43]. The dependent variables were represented by (1) number of eggs collected in the ovitraps (OVTs), (2) in the double BR-ovts and (3) total number of eggs monitored in the two types of ovitraps (TOTAL). Categorical predictors were represented by type of group (single/double) and cycles (monitoring period every 28 days). On the other hand, the continuous predictor was represented by the total number of adult insects. A general linear model (GLM) was used to assess the difference between groups, assessing the effect of each predictor and the interaction between categorical predictors. In addition, multiple regressions were performed to verify the existence of a relationship between the dependent variables and climatic factors, such as precipitation and maximum temperature. Regressions were also performed between the total number of adult insects and the same climatic factors, for the different groups. The normality of data residues and homoscedasticity were verified by Shapiro–Wilk and Levene tests, respectively. All analysis was performed using the Statistic 13.0 program.

## 3. Results

### 3.1. Integrated Surveillance and Control Actions

**Figure 3:**
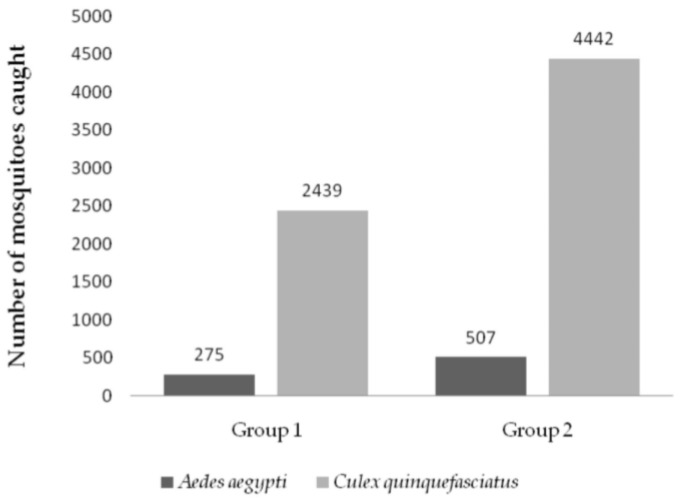
Number of mosquitoes captured in Nova Descoberta neighborhood (*n* = 40 properties). Group 1: simple control actions (*n* = 20 properties); group 2: double control actions (*n* = 20 properties). Data collected from January to December 2018 (cycles 1 to 12) through mechanical aspiration in the project’s properties.

The control actions used in the different groups of this study (single and double) removed a high amount of eggs and adult mosquitoes from the environment. A total of 7663 mosquitoes were collected; 6881 (89.7%) of them were specimens of *Cx. quinquefasciatus* and 782 (10.2%) specimens of *Ae. aegypti* (Figure 3). For the analysis, we considered only indoor samples due to the lower number of mosquitoes (<5%) captured in outdoor places, and the majority of them were male. In all cycles of the study, the *Cx. quinquefasciatus* was predominant in adult mosquitoes captured by mechanical aspiration. No other species of mosquito were caught. Sometimes, other insects were present but were discarded from the analysis.

**Figure 4:**
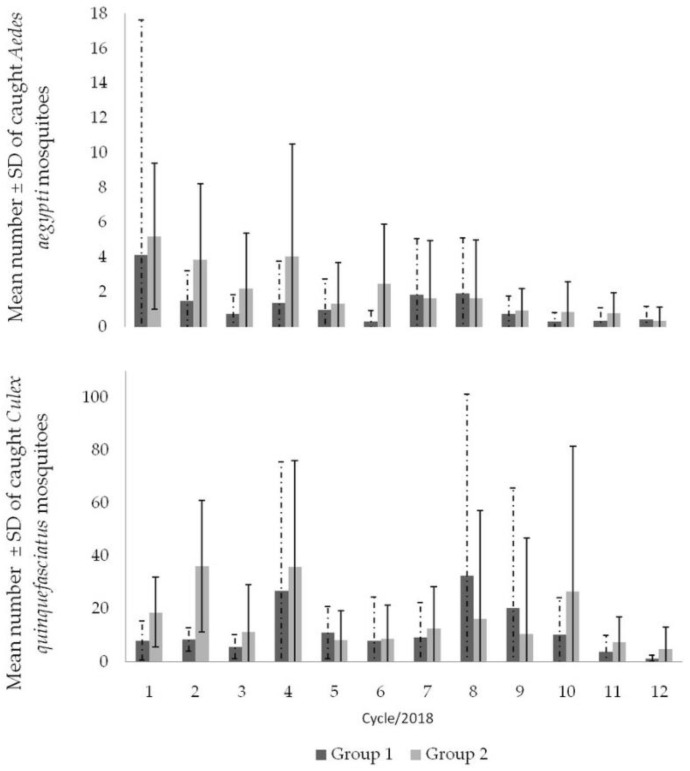
Mean number of mosquitoes caught per cycle in the different groups evaluated in Nova Descoberta. Data collected from January to December 2018 (cycles 1 to 12). Group 1: simple control actions (*n* = 20 properties); group 2: double control actions (*n* = 20 properties).

Groups 1 and 2 showed different densities of *Ae. aegypti*, but a non-significant difference between them, with an average number of 1.3 ± 4.8 mosquitoes collected for group 1 and 2.2 ± 3.6 for group 2. Analyzing the collection of adults of *Ae. aegypti* over the 12 cycles, in the two groups, subtle fluctuations are noticeable, but without significant differences between cycles (F: 2.97; *p* > 0.05), despite a reduction in the average number of adults collected (96.1%) (Figure 4). For *Cx. quinquefasciatus*, similar to *Ae. aegypti*, the groups showed no statistical differences (F: 2.97; *p* > 0.05), with an average of 11.6 ± 28.4 mosquitoes/cycle/property collected in group 1 and 18.8 ± 30.8 mosquitoes/cycle/property in group 2 (Figure 3). The study groups showed a 77.1% reduction in *Cx. quinquefasciatus*. (Figure 3).

**Figure 5:**
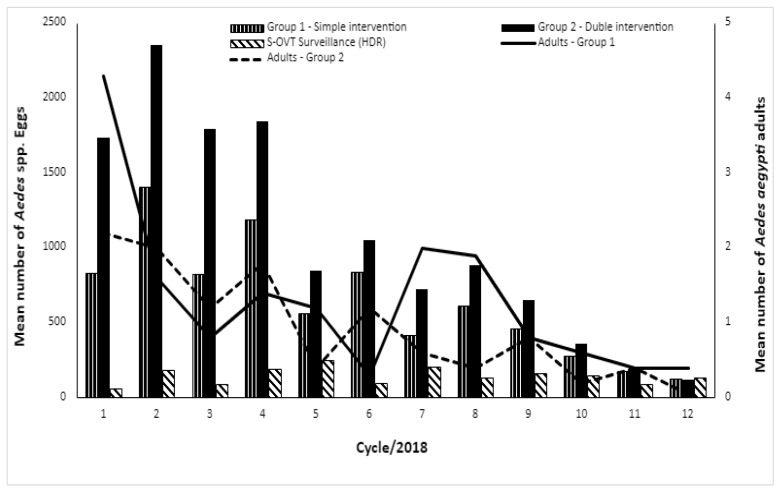
Mean number of Aedes spp. eggs collected through oviposition traps (OVT), Double BR-OVT and Sentinel-Ovitrap (S-OVT), and mean number of *Aedes aegypti* adults captured by indoor aspiration. Group 1: simple control actions (*n* = 20 properties) and group 2: double control actions (*n* = 20 properties). Data collected from January to December 2018 (cycles 1 to 12). Source: entomological surveillance data from Health Department of Recife.

The use of TSBs attached in the Double BR-ovt and OVT resulted in the death of many adult mosquitoes on and near the baits. Although it was not possible to count the mosquitoes due to the degradation of the material after 28 days, the visualization of the dead mosquitoes suggests its effectiveness. Another result that showed a complementary control role of the TSBs, especially for the elimination of *Ae. aegypti*, was detected in the adult density throughout the cycles 7 to 12, when the mean number of mosquitoes dropped progressively until a range of 0.05 mosquito/property/cycle (Figure 5; Table 1). Although the use of TSBs with the Double BR-ovt and OVT promoted a reduction in the mosquito density compared to the traps without it, once more, the difference between them was not significant (*p* > 0.05).

The number of properties evaluated throughout the study decreased, from three to nine properties in group 1 and a maximum of two properties in group 2 due to properties being closed on operator visit days (Table 1). Regarding the number of properties investigated in group 1, less than 10 of them were positive for *Ae. aegypti* and more than 14 properties were positive for *Cx. quinquefasciatus*. This characteristic was also observed in group 2. However, for *Ae. Aegypti*, it was reduced to six properties in the second half of the year, when TSBs were added to the control scheme, except in September 2018. In the last evaluation cycle (December 2018), only one property in this group was positive for the same species. The interventions led to a cumulative reduction of 90% and 98% in groups 1 and 2, respectively, after one year of control. These results reveal the impact of joint actions on the elimination of adult *Ae. aegypti* and, consequently, the reduction in mosquito–human contact, also mentioned by residents of the properties studied. The same control effectiveness was not registered for *Cx. quinquefasciatus*, although there was a reduction in the population density of adults when comparing groups 1 and 2 in the second semester. In addition, the presence of TSBs combined with traps prevented the explosive population growth observed in August and September 2018 (Table 1 and Figure 5).

**Figure 6:**
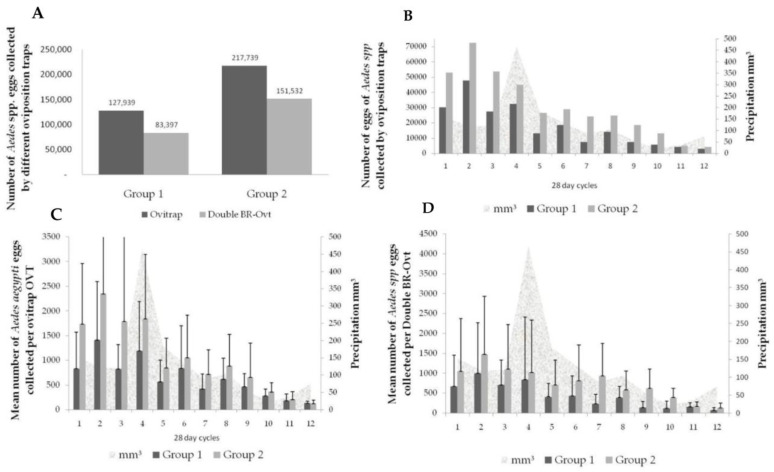
The total number (**A**,**B**) of Aedes eggs collected by different oviposition traps in the different evaluated groups. Data collected from January to December 2018 (cycles 1 to 12). Mean number of eggs collected through oviposition traps (OVTs) (**C**) and Double BR-ovt. Group 1: simple control actions (*n* = 20 properties) and group 2: double control actions (*n* = 20 properties) (**D**).

Oviposition traps allowed for a massive removal of eggs from the environment, exceeding half a million eggs captured for both trap models (Figure 6). The OVT remained positive throughout the 12 evaluation cycles, where we found a minimum quantity of 3 eggs and a maximum of 8758 eggs/with 2 straws; 1 to 3859 eggs/OVT with cotton fabric and 2 to 4664 eggs/Double BR-ovt, also lined with fabric. It was possible to notice a significant difference in the number of *Ae. aegypti* collected between cycles (F = 10.77; gL. 11; *p* < 0.00001). Furthermore, in group 2, there was a significantly greater collection of eggs (F = 14.92; gL. 1; *p* < 0.005) when compared to group 1 (Figure 6). The egg density decreased gradually until 90%, regardless of the group evaluated after the 12 cycles. In group 1, there was a reduction from 1433.5 ± 1108.3 to 142.3 ± 121.6 and in group 2, a reduction from 2785.5 ± 2273.6 to 215.7 ± 152.5. (Figure 6). The dispersion of the *Ae. aegypti* regardless of the group evaluated remained at 100%, although many traps were positive with only a few eggs per month. 

Although we observed similarity between the precipitation fluctuation and the number of eggs or adult mosquitoes captured, for both groups, the statistical analysis did not reveal a positive relationship between these variables (*p* > 0.05).

A significant reduction in the number of eggs collected by the OVT and Double BR-ovt traps in the second semester of 2018 was observed, while a different behavior was observed in the S-OVT used for entomological surveillance by the Health Secretary of Recife in Nova Descoberta (Figure 4). This result demonstrates that only those interventions conducted by the present study were effective.

### 3.2. Vectorial Infection Detection

During the 12 study cycles, a total of 8496 females of *Ae. aegypti* and *Cx. quinquefasciatus* were stored in 1638 pools at −80 °C for the molecular investigation of the arbovirus vectorial infection (Zika, Chikungunya and Dengue). When evaluating the vector infection by ZIKV and DENV (220 pools), 19% of pools from both *Ae. aegypti* and *Cx. quinquefasciatus* were positive for arboviruses. For *Ae. aegypti*, 100 pools (225 ♀) were analyzed, of which 29% were infected. The highest rates of vectorial infection (Table 2) of *Ae. aegypti* with ZIKV were found in April (MIR—214.3), August (MIR—230.7) and September (MIR—411.7)—2018. The study area presented an annual MIR of 128.8, with 29 positive pools among 225 females analyzed. *Culex quinquefasciatus* females were analyzed through 120 pools (721 ♀), showing 10% of infected pools for ZIKV. The highest infection rates of this species with ZIKV occurred in January (MIR—75.9), February (MIR—40.5) and October (MIR—36.3)—2018 (Table 2). Regarding vectorial infection by DENV, among the 100 pools of *Ae. aegypti*, only 1 positive pool was detected. This particular sample was collected in July 2018. The annual MIR for DENV was 4.4. Among the samples from female *Cx quinquefasciatus* analyzed, no positive samples for CHIKV were detected.

## 4. Discussion

During this study, we found that the use of better and integrated strategies can result in a positive impact on the control of *Aedes aegypti* and *Culex quinquefasciatus* mosquito populations. More than half a million eggs of *Aedes* spp. were removed from the environment throughout one single year, with approximately 120 traps. The OVT and Double BR-ovt remained permanently positive in the field. These results agree with another study carried out in Recife [32] (Regis et al., 2008), in 2004, using around 3600 OVT-Cs, house to house, for five consecutive months, in the neighborhood of Engenho do Meio, where more than 6 million eggs were eliminated. In both studies, the results show a strong colonization pressure by *Ae. aegypti* and highlight the continuous presence of blood-fed females in properties.

The use of different tools to control mosquitoes in all development phases contributed to a progressive and sustainable reduction in the evaluated entomological indices, adult Done.and egg densities, compared to the indices initially observed in each group, despite the high and constant positivity (100%) of the oviposition traps. In addition, the circulation of arboviruses (ZIKV and DENV) detected in 19.5% of the analyzed mosquitoes reinforces the importance of this type of study and the maintenance of permanent entomological surveillance in tropical regions such as Northeast Brazil. Intervention plans adopted in the properties for both groups were successful in greatly reducing the contact of adult mosquitoes with the human residents, especially to *Ae. aegypti* (>99%), in one year. We demonstrated here that integrated control actions, even when adopted exclusively in isolated properties, can reduce the population densities of these hematophagous insects, and it can be used as a continuous protection strategy for people with greater vulnerability to arbovirus infection, in the smallest territorial scale of epidemiological risk, that is, the home. Our data corroborate the study carried out by Barrera et al. [26], which evaluated control actions on *Ae. aegypti* (source reduction, larvicide, sticky traps) in target properties in Puerto Rico, achieving a reduction of 92, 4% in the egg density. Our results demonstrated the dual advantage of oviposition traps as a strategic tool: the first one related to the withdrawal of viable eggs, and the second one reduces, in the long-term, the quiescent eggs’ storage in the environment, avoiding the recompositing of the *Ae. aegypti* population in treated areas, as frequently observed by the PNCD program. The particular intervention implemented in group 2 was designed to test if the intensification of these actions would bring an improvement in quickly reducing the densities of *Ae. aegypti*. Our results showed that the intensification of actions significantly expanded (*p* < 0.05) the spectrum of the collection of eggs and mosquitoes, and reduced the number of infested properties, suggesting a potential decrease in the spread of arbovirus transmission. However, the continuous pressure to simultaneously remove adult mosquitoes and eggs from the environment, and the strategies employed (single or double), showed similar responses at the end. The intensification of actions carried out in group 2 proved to be an interesting strategy to be used in the initial phase of the plan to control mosquitoes or in the first semester of the year, in local conditions, when a large number of arbovirus cases are expected. However, when densities reach manageable levels, the field effort can be reduced by using fewer capture tools and more TSBs and control traps such as ovitrap and double BR-OVT.

We emphasize that this area is classified as high risk for arboviruses and has characteristics that increase the possibilities of real and potential breeding sites for the maintenance of these mosquitoes in the environment (urban agglomerations, low coverage of basic sanitation and intermittent water supply). We commonly found potential breeding sites in the properties, with preferential characteristics for *Ae. aegypti*, such as water storage (buckets, water tanks, cisterns, pots) or other artificial breeding sites (large open channels, culverts and grease traps), with characteristics considered preferable for the *Cx. Quinquefasciatus* species, as they accumulate organic matter. These data demonstrate how particular conditions in the micro-level can influence the maintenance of mosquito populations. The breeding sites were treated with Vectomax^®^, manufactured by Valent BioSciences technical, Libertyville, IL, USA, a conjugated biolarvicide (Lsp + Bti), that represents operational advantages and a broad spectrum of action, eliminating larvae of both *Ae. aegypti* and *Cx. quinquefasciatus,* with a lower risk of resistance selection [35,44,45].

Through the oviposition traps, it was possible to carry out a massive capture of *Aedes* eggs in the neighborhood (580, 607 eggs), but we do not have any estimation regarding how many *Cx. quinquefasciatus* eggs were eliminated in the same period. More than this, the scheme of interventions adopted inside of the isolated properties did not have the same impact for *Cx. quinquefasciatus*, since we can see a decrease in their density (<70%) but not in their distribution in the studied properties, once the OPI was 100% in the traps evaluated in every cycle. These results showed that for this species, the breeding sites beyond the properties are determinants to sustain their infestation, more than for *Ae. aegypti*. We believe that the uninterrupted permanence of traps throughout the evaluation period may have favored the high maintenance of this index, in addition to the already known high density of the species in the area. Other studies have also demonstrated the traps’ positivity above 90%, indicating a very high degree of sensitivity in ovitrap monitoring [45,46,47]. Regarding the density of eggs removed from the environment, results just like ours have been observed by other authors [32,48]. Several studies, performed in different regions, demonstrated a greater sensitivity for detecting the *Ae. aegypti* species using an ovitrap compared to the use of other tools, such as larval survey [32,49].

Our study used the Double BR-ovt trap for *Aedes aegypti* and *Culex quinquefasciatus* [28] as a monitoring and control tool. We performed a small modification with the removal of the adhesive edge to avoid the capture of adult mosquitoes. This trap proved to be as efficient in collecting eggs as the OVT installed in the outdoor area and is widely used in the world. Xavier et al. [28] demonstrated the potential of a Double BR-ovt in mosquito monitoring strategies by conducting studies in Olinda (Pernambuco—Brazil), collecting an average of 410 ± 588.3 eggs from the *Aedes*/property/cycle, showing that the female can search for attractive indoor breeding sites [29]. We also found that the use of two Double BR-ovt traps installed indoors, combined with adults’ mechanical aspiration, increases the number of mosquitoes collected in resting places, as indicated by Xavier et al. [28]. Behavioral characteristics of these insects, such as anthropophilia and endophilia, favor the success of this trap. Other studies have observed that female mosquitoes prefer the home environment to rest and for a blood meal [15,50]. A study by Barata [51], in São José do Rio Preto (São Paulo, SP, Brazil), an endemic area for dengue, found that of the 189 adult females of *Ae. aegypti* captured, 87.3% were indoors. These data demonstrate that indoor control actions, such as the installation of Double BR-ovt traps associated with ITA, are important for reducing the population density of *Ae. aegypti* and *Cx. quinquefasciatus*.

Mechanical aspiration performed indoors allowed for the removal of more than 7000 adult mosquitoes from the study area, predominantly collecting the *Cx. quinquefasciatus* species (89.7%). This fact may be related to a collection period favorable to the capture of this species, which is at rest, different from *Ae. aegypti*, a diurnal species that usually search for blood feeding at this time, which can be difficult to catch. According to Regis et al. [26], the aspiration of adult mosquitoes is an effective tool in monitoring species and, if used intensively, it can also have a population control action. Another tool used in our study that contributed to reducing the adult density was toxic sugar bait (TSB) [39,52,53]. We consider that the TSB attached to the oviposition traps improved the control of adult mosquitoes by acting as an available source of a lethal carbohydrate meal in the same places where females chose to lay eggs. In addition, residents reported many dead mosquitoes inside or around the traps and a decreased harassment of mosquito bites after the TSB installation. However, we could not see and count the dead mosquitoes due to their degradation throughout the time. More than this, we believe that most mosquitoes fed on TSB suffered the lethal effect later, reaching far more mosquitoes than those seen dead inside or near to the TSB, as reported by other studies [39,52,53]. Our tests in the laboratory showed a significant mosquito mortality registered until 24 h after the ingestion of the TSB (unpublished data), corroborating the Tenywa et al. [39] observation of using a sugary meal containing 0.005% ivermectin to control *Anopheles arabiensis* (Patton, 1905).

This study proposes to include the analysis of vector infection in mosquitoes as a complementary strategy for viral surveillance and control programs, thus enabling the tracking of the presence of an arbovirus in the environment. Even after the considerable decrease in the mosquito population size, we were able to detect ZIKV via RT-qPCR in a pool sample consisting of a single female *Ae. aegypti*. Nova Descoberta is considered an endemic area for dengue, Zika and chikungunya; among the 898 females analyzed from that location, 20% of the pools were infected with ZIKV. We did not obtain any samples infected with CHIKV, and only one sample was positive for DENV. The detection of mosquitoes, mostly infected with ZIKV, was an unexpected result, once Recife Health Epidemiological Surveillance bulletins did not point to the wide circulation of dengue and Zika throughout 2018 in the study area. In addition, there was an increase in the number of dengue cases across Pernambuco state at the end of 2018 and throughout 2019, including Recife [13]. The role of *Ae. aegypti* in the ZIKV transmission cycle is already well known and studied, this species being considered the main vector of the virus in the urban environment [54]. Nonetheless, the participation of *Cx. quinquefasciatus* as a potential ZIKV vector is still much discussed worldwide. Two studies, conducted in Brazil and China, demonstrated the ability of *Cx. quinquefasciatus* to transmit ZIKV [55,56]. Other studies have shown that many geographically distinct populations are refractory to virus transmission [57,58,59]. Although we found ZIKV in the two main species of mosquitoes present in the study area, the ZIKV infection rate in *Cx. quinquefasciatus* (18.33) was much lower when compared to *Ae. aegypti* (153.43).

These data suggest that *Culex quinquefasciatus*, even though many authors do not consider it a ZIKV vector, may participate as a secondary vector contributing to the viral cycle, which underscores a special attention concerning the control of the Zika fever disease. Studies carried out in Malaysia and Thailand captured mosquitoes in the domiciles of ZIKV-infected people or in the surrounding area and detected the presence of the virus in mosquitoes of the *Aedes* and *Cx. quinquefasciatus* genus, indicating that they could be potential vectors [60,61,62].

Throughout the investigation cycles, around 50% of the properties evaluated showed pools of mosquitoes infected by ZIKV and, of these, 27% were repeated for more than three to five cycles (consecutive or close). Using tools that evaluate the density of mosquitoes and the detection of vector infection, we noticed that the properties with the highest number of eggs and adult densities were also those that had the most repetitions of infected samples. According to a study carried out in Italy by Calzolari et al. [62], which analyzed the infection of *Cx. quinquefasciatus* with West Nile Virus, the number of infected mosquitoes increases when the number of mosquitoes also increases. When comparing our molecular data with the Zika fever notification data in the studied areas, we noticed a high percentage of vector infection detection, despite the absence of notifications from sick people in the evaluated properties and nearby, suggesting that the disease was likely being underreported. This may occur due to inconsistencies in differential diagnostic tests, which are often not available at health centers, and the occurrence of asymptomatic and mild infections [63]. Some studies have already shown that asymptomatic ZIKV infection is common after periods of outbreak when there is an increase in the population’s immune resistance to the virus and in pregnant women [63,64,65]. Other authors also support the argument that the transovarial transmission of ZIKV occurs in *Ae. aegypti* and *Cx. quinquefasciatus* in the field [66,67]. Continuous surveillance of circulating strains of arboviruses is of utmost importance once epidemic and inter-epidemic transmission in the vector mosquito to human population suggests that arboviruses will continue to evolve.

Surveillance and control efforts must be strengthened and coordinated on a large scale, as currently, no global surveillance system is in place to track the emergence and spread of mosquito-borne diseases. The monitoring, control and diagnosis of vector infection allowed us to trace the profile of local infestation and to suggest a plan for the surveillance and control of the population density of mosquitoes, especially for underdeveloped areas of tropical regions that have high temperatures and humidity, in addition to fragile socioeconomic conditions and environmental factors. This study demonstrates that it is possible to reduce mosquito densities locally by working in target properties, with surveillance and control actions, using low-cost and easy to use tools. In addition, these tools allow for the surveillance of arboviruses circulating in vector mosquitoes. We assess that the community’s participation and interest in acquiring knowledge and acting in the fight against mosquitoes that carry diseases that affect the population suggest new possibilities for strategies for the surveillance and control of these insects. We achieved a gradual and sustained reduction in the evaluated entomological indexes (AD and ED) and detected the circulation of the ZIKV and DENV arboviruses in the studied area.

## 5. Conclusions

The reduction in the density of *Ae. aegypti* and *Cx. quinquefasciatus* observed in properties heavily infested by these mosquitoes, in a neighborhood in the city of Recife, was possibly favored by the use of integrated strategies to simultaneously eliminate eggs and adults. The intensification of the use of oviposition traps, aspiration and elimination of adult mosquitoes by TSBs has led to a sustainable reduction in mosquito–human contact in areas with an active circulation of arboviruses. Although we did not observe statistical differences in the reduction in the adult density in the different groups, for *Aedes* eggs, statistical differences were observed between them. Therefore, implementing improved control actions using some low-cost tools will bring satisfactory results with less effort. Therefore, we recommend that the strategy of intensifying control actions, here applied to group 2, be used in the initial phase of control programs or in periods in which the large population growth of *Ae aegypti* and/or *Cx. quinquefasciatus* is expected. This study brings the first use of TSBs to control *Ae. aegypti* in Brazil. We conclude that it is extremely important to include the surveillance of vector infections in mosquito control programs, as it allows for the detection of the cryptic circulation of arboviruses before outbreaks occur. Early detection of arbovirus vector infections, including Zika, can guide interventions in priority areas and minimize the consequences of the disease on affected children and families.

## Figures and Tables

**Table 1:** Monitoring of parameters associated with the evaluation of intervention plans adopted in group 1: simple control actions (*n* = 20 properties) and group 2: double control actions (*n* = 20 properties), on adult mosquitoes of the *Aedes aegypti* and *Culex quinquefasciatus* species. Data collected from January to December 2018 (cycles 1 to 12).

Intervention Plans/Species	Monitoring Cycle of Adult Mosquitoes											
	1	2	3	4	5	6	7	8	9	10	11	12
Single Actions Group 1												
*Aedes aegypti*												
N of properties	20	17	17	16	11	15	14	16	16	16	16	19
N of positive properties	12	12	7	11	5	4	8	10	7	6	4	6
Total number of mosquitoes	86	27	13	22	13	5	28	31	12	5	6	8
Mean number	4.3	1.6	0.8	1.4	1.2	0.3	2.0	1.9	0.8	0.3	0.4	0.4
Standard Deviation	13.9	1.7	1.1	2.4	1.8	0.6	3.3	3.2	1	1.3	0.7	0.8
*Culex quinquefasciatus*												
N of properties	20	17	17	16	11	15	14	16	16	16	16	19
N of positive properties	17	16	17	15	11	11	11	15	14	14	7	14
Total number of mosquitoes	168	145	98	432	124	122	137	520	327	155	64	26
Mean number	8.4	8.5	5.8	27.0	11.3	8.1	9.8	32.5	20.4	9.7	4.0	1.4
Standard Deviation	7.4	4.5	4.5	48.6	9.7	16.3	13.7	68.7	45.2	38.6	6.2	1.3
Double Actions Group 2												
*Aedes aegypti*												
N of properties	20	20	20	20	20	20	20	18	19	19	19	19
N of positive properties	18	16	10	11	6	11	8	5	11	3	6	1
Total number of mosquitoes	42	37	23	34	8	23	11	6	13	4	7	1
Mean number	2.1	1.9	1.2	1.7	0.4	1.2	0.6	0.3	0.7	0.2	0.4	0.05
Standard Deviation	1.5	1.9	1.4	2.2	0.7	1.4	0.9	0.6	0.7	0.5	0.6	0.2
*Culex quinquefasciatus*												
N of properties	20	20	20	20	20	20	20	18	19	19	19	19
N of positive properties	19	19	16	19	14	15	16	15	16	19	16	15
Total number of mosquitoes	99	148	95	225	58	103	83	133	104	120	57	43
Mean number	5.0	7.4	4.8	11.3	2.9	5.2	4.2	7.4	5.5	6.3	3.0	2.3
Standard Deviation	4.0	5.6	5.7	9.1	4.2	5.1	4.7	7.1	7.7	8.6	3.6	3.5

Mean number of mosquitoes collected through aspiration.

**Table 2:** Pools of up to 10 females of *Aedes aegypti* and *Culex quinquefasciatus* captured by entomological aspirator within and around the house, analyzed for Zika infection in the study area. Data collected from January to December 2018 (cycles 1 to 12).

Species	MONTH 2018																							
	January				February				March				April				May				June			
	♀	NP	P+	MIR	♀	NP	P+	MIR	♀	NP	P+	MIR	♀	NP	P+	MIR	♀	NP	P+	MIR	♀	NP	P+	MIR
*Ae. aegypti*	24	10	0	0	21	10	0	0	16	10	0	0	28	10	6	214.2	17	7	3	176.4	27	10	5	185.2
*Cx. quinquefasciatus*	79	10	6	76.0	74	10	3	40.5	65	10	0	0	72	10	0	0	70	10	0	0	71	10	0	0
	**July**				**August**				**September**				**October**				**November**				**December**			
	♀	NP	P+	MIR	♀	NP	P+	MIR	♀	NP	P+	MIR	♀	NP	P+	MIR	♀	NP	P+	MIR	♀	NP	P+	MIR
*A aegypti*	24	10	0	0	26	10	6	230.7	17	10	7	411.7	14	6	0	0	11	7	2	182	0	0	0	0
*Cx. quinquefasciatus*	67	10	0	0	54	10	0	0	32	10	0	0	55	10	2	36.36	49	10	0	0	33	10	1	30.3

Note: ♀—number of females analyzed; NP—number of pools of females analyzed; P+—number of pools with females infected; MIR—Minimum Infection Rate.

## Data Availability

Data are contained within the article and supplementary materials. Data are contained within the article and supplementary materials.

## Supplementary Materials

The following supporting information can be downloaded at: https://www.mdpi.com/article/10.3390/tropicalmed9030053/s1, Table S1: description of the reagents used for samples of *Aedes aegypti* and *Culex quinquefasciatus* in the RT-qPCR *singleplex* reaction for ZIKV detection; Table S2: description of the reagents used for samples of *Aedes aegypti* in the RT-qPCR *duplex* reaction for CHIKV and DENV detection and Table S3: description of the primers and probes used in the RT-qPCR reactions.
