# Supplementary material for: Integrated Strategies for Aedes aegypti Control Applied to Individual Houses: An Approach to Mitigate Vectorial Arbovirus Transmission
Source: Trop Med Infect Dis. 2024 Feb 24;9(3):53. doi: 10.3390/tropicalmed9030053 (PMC10974253; doi:10.3390/tropicalmed9030053)
Supplement: Supplementary file 1 [file tropicalmed-09-00053-s001.zip › tropicalmed-2662119-supplementary.pdf]

Tropical Medicine and Infectious Disease

Article

Integrated Strategies for *Aedes aegypti* Control Applied to Individual Houses: An Approach to Mitigate Vectorial Arbovirus Transmission

Table S1. Description of the reagents used for samples of *Aedes aegypti* and *Culex quinquefasciatus* in the RT-qPCR *singleplex* reaction for ZIKV detection.

Reagent	Concentration	Measure (µL)
**H2O**	-	1,15
**QuantiNova Probe RT-PCR Master Mix**	2x	5,0
**Primer ZIKV1163c**	100 µM	0,08
**Primer ZIKV1087**	100 µM	0,08
**Probe FAM - ZIKV**	25 µM	0,04
**QuantiNova ROX™**	200 x	0,05
**QuantiNova RT Mix**	100 x	0,1
**RNA**		3,5
**Final measure**	-	10

Table S2. - Description of the reagents used for samples of *Aedes aegypti* in the RT-qPCR *duplex* reaction for CHIKV and DENV detection. .

Reagent	Concentration	Measure (µL)
**H2O**	-	0,95
**QuantiNova Probe RT-PCR Master Mix**	2x	5,0
**Primer CHIKV 6856**	100 µM	0,08
**Primer CHIKV 6981**	100 µM	0,08
**Probe VIC - CHIKV**	25 µM	0,04
**Primer DENV G Fw**	100 µM	0,08
**Primer DENV G Rv**	100 µM	0,08
**Probe Cy5 - DENV**	25 µM	0,04
**QuantiNova ROX™**	200 x	0,05
**QuantiNova RT Mix**	100 x	0,1
**ARN**		3,5
**Final measure**	-	10

Table S3. - Description of the primers and probes used in the RT-qPCR reactions.

Primer/Probe	5′ – 3′
**Primer ZIKV1163c**	CCACTAACGTTCTTTTGCAGACAT
**Primer ZIKV1087**	CCGCTGCCCAACACAAG
**Primer CHIKV 6856**	TCA CTC CCT GTT GGA CTT GAT AGA
**Primer CHIKV 6981**	TTG ACG AGA GTT AGG AAC ATA CC
**Primer DENV G Fw**	AAGGACTAGAGGTTAGAGGAGACCC
**Primer DENV G Rv**	CGTTCTGTGCCTGGAATGATG
**Probe FAM – ZIKV**	AGCCTACCTTGACAAGCAGTCAGACACTCAA
**Probe VIC - CHIKV**	AGGTACGCGCTTCAAGTTCGGCG
**Probe Cy5 - DENV**	AACAGCATATTGACGCTGGGAGAGACCAGA
